# Metagenomic profiling of bacterial (16S) and fungal (ITS) communities on d’Anjou pears during long-term controlled-atmosphere storage

**DOI:** 10.1128/spectrum.04117-25

**Published:** 2026-05-18

**Authors:** Rawane Raad, Amy Mann, Amrit Pal, Angela Parra, Laura Strawn, Alexis Hamilton, Faith Critzer, Henk C. den Bakker

**Affiliations:** 1 Department of Food Science and Technology, University of Georgia, Athens, Georgia, USA; 2 Center for Food Safety, University of Georgia, Griffin, Georgia, USA; 3 Department of Food Science and Technology, Virginia Tech 1757, Blacksburg, Virginia, USA; University of Mississippi, University, Mississippi, USA

**Keywords:** pear, microbiome, controlled-atmosphere, postharvest, tree fruit

## Abstract

D’Anjou pears are routinely stored for up to nine months under controlled-atmosphere (CA) conditions to meet market demands. While this practice maintains fruit quality, limited information exists on pears’ natural microbiota throughout storage. The objective of this study was to describe fungal and bacterial composition on marketable and unmarketable conventional, whole, intact pears under two storage practices (bulk vs wrapped) at 3, 6, and 9 months in long-term CA cold storage. Storage practices had a significant effect on the composition and succession of both fungal and bacterial communities. No significant differences in Chao1 index were found between the bacterial and fungal communities on marketable or unmarketable pears. Trends in Chao1 indices of fungal and bacterial communities peaked at mid-storage and declined by 9 months, with wrapped pears showing parallel trends, and bulk pears exhibiting a sharper late-stage reduction. No distinct clusters could be found for 3- and 6-month fungal communities, irrespective of marketability, or whether bulk or wrapped. The principal coordinate analysis of the bacterial communities showed tight clustering by time point for the individually wrapped pears, irrespective of their marketability. Bacterial communities included genera common in food-processing and plant environments, such as *Pseudomonas* (19.2% relative abundance [RA]) and *Acinetobacter* (3.31% RA). Fungal communities shifted over time, with spoilage-associated genera like *Aureobasidium* (23.3% RA), *Penicillium* (9.28% RA), *Botrytis* (0.33% RA), and *Mucor* (0.14% RA) present at different storage stages.

**IMPORTANCE:**

This study highlights the influence of storage duration and packaging on microbial succession, establishing initial benchmarks of pear surface microbiomes. The observed lack of significant differences in microbial diversity between marketable and unmarketable pears suggests that these baseline community profiles can serve as critical reference points for identifying other influential factors. Variables such as handling practices may exert a more direct effect on microbial dynamics and, consequently, product quality. Establishing these baselines is essential because they provide a foundation for detecting deviations linked to spoilage or safety risks. Moreover, understanding these patterns can guide the development of targeted microbial control strategies in postharvest systems, enabling interventions that maintain fruit quality, reduce losses, and possibly improve food safety throughout the supply chain.

## INTRODUCTION

Pear (*Pyrus* spp.) production in the United States contributes to 84% of the national fresh pear crop consumption (1). Production is largely concentrated in the Pacific Northwest (Washington State, Oregon, and California), where the industry generates over $250 million in annual sales (1). The three main cultivars grown in this region are d’Anjou, Bartlett, and Bosc, with d’Anjou pears representing the largest acreage and highest economic return for U.S. growers (1, 2). To meet year-round market demands and ensure consistent supply, pears must be stored for an extended period of time after harvest. Long-term storage allows growers and distributors to manage seasonal fluctuations in production, maintain fruit availability in off-season markets, and reduce postharvest losses. Controlled-atmosphere (CA) storage, which regulates oxygen, carbon dioxide, and temperature, has become the industry standard for preserving fruit quality over several months in pears by slowing physiological and microbial processes. CA environments typically maintain low oxygen (2–5 kPa O₂) and elevated carbon dioxide levels under cold temperatures, which suppress respiration, reduce ethylene production, and delay senescence (3, 4). These conditions help preserve tissue integrity and indirectly limit microbial spoilage by reducing opportunities for pathogen invasion. Effective long-term storage is a critical component of commercial pear production. It not only maintains firmness, flavor, and nutritional value but also minimizes economic losses associated with spoilage and decay. Common practices throughout storage include storing the pears in bulk in plastic bins or wrapping each pear with tissue paper impregnated with copper carbonate, food-grade oil, and/or antioxidants such as ethoxyquin to prevent postharvest disorders and the spread of fungal decay organisms (5, 6). These different conditions may play a key role in the microbiota that are present on pear surfaces and how they may change throughout storage duration.

Fruit surfaces host diverse bacterial and fungal communities, collectively known as the epiphytes (7). These microbial communities play important roles in postharvest quality, influencing fruit spoilage, shelf life, and, in some cases, potential food safety risks. Although no major foodborne outbreaks have been directly associated with pears, other tree fruits such as apples (8) and peaches (9) have been implicated in contamination events, underscoring the need to understand and monitor the microbial populations on pear surfaces. High-throughput sequencing approaches have revealed that fruit surfaces harbor diverse bacterial and fungal taxa beyond what traditional culturing methods can detect, emphasizing the need to consider both culturable fractions and the total microbiota when assessing microbial ecology. Understanding the fruit microbiome opens new avenues for postharvest biocontrol strategies, shifting the paradigm from targeting single pathogens to managing entire microbial consortia (7). For example, Abdelfattah et al. (10) demonstrated that common postharvest practices such as washing, waxing, and cold storage significantly reshape the apple microbiome, with implications for pathogen suppression and quality maintenance. Al Riachy et al. (11) further showed that long-term storage alters the epiphytic microbiome of apples and influences *Penicillium expansum* occurrence and patulin accumulation, underscoring the dynamic nature of these communities under commercial conditions. Collectively, these findings inform industry practices by suggesting that interventions should account for microbiome shifts over time and under different storage regimes.

Historically, studies of produce-associated microorganisms relied on culture-based methods, which remain the regulatory gold standard for detecting viable pathogens and allow functional characterization of isolates (12). However, these approaches are inherently limited by selective growth conditions and often underestimate microbial diversity, as many taxa are unculturable or require specific growth factors not replicated *in vitro* (13, 14). In contrast, sequencing-based techniques such as 16S rRNA and ITS amplicon sequencing have revolutionized our understanding of the total microbiota, revealing complex communities that include both dominant and rare taxa (10, 11, 15). Culture-based studies still dominate regulatory testing and targeted pathogen isolation, but sequencing approaches have become the primary tool for hypothesis generation and ecological assessment. This trend underscores a broader movement in food microbiology toward multi-modal strategies that combine culturing and molecular tools to design interventions that maintain fruit quality and safety throughout the supply chain.

Despite the widespread use of CA storage in the pear industry, there is limited information on how such conditions affect the surface microbiome of pears over extended periods. Understanding these potential successional changes in both bacterial and fungal communities is critical for predicting how the microbiome evolves during storage and how it may impact fruit quality. Advances in molecular techniques, including 16S rRNA gene sequencing for bacteria and ITS sequencing for fungi, allow the characterization of these communities (16). Compared to traditional culture-based methods, these approaches capture slow-growing, stressed, or otherwise unculturable taxa, providing a more complete view of the pear surface. It is also important to acknowledge that DNA-based microbiome approaches detect both viable and non-viable microorganisms, meaning that some taxa identified may represent residual DNA from dead cells rather than active members of the community.

Previous research on other fruit commodities has increasingly focused on characterizing the complex microbial communities associated with fresh produce and postharvest environments, as these microbiomes influence product quality, safety, and shelf life (11). For example, Bosch et al. (17) demonstrated that controlled-atmosphere storage alters microbial community structure in apples, while others (18, 19) reported viable bacterial populations and pathogen persistence on pears under CA conditions. Our study expands this landscape by characterizing both bacterial and fungal communities on pears under long-term CA storage and assessing how packaging and marketability interact with storage duration to shape these communities. This study was initiated at the request of industry stakeholders seeking microbiome-informed guidance on packaging and quality management during extended storage. We therefore set out to profile bacterial (16S rRNA V3–V4) and fungal (ITS1) communities across storage time and to test how packaging format (individually wrapped vs. bulk) and marketability status (marketable vs. unmarketable), with marketability serving as a proxy for differences in fruit surface condition (e.g., minor defects, handling stress, or cuticle integrity) that may influence microbial colonization and persistence, relate to alpha and beta diversity. We hypothesized that (i) storage time would influence microbial diversity and composition due to prolonged environmental exposure, (ii) packaging type (wrapped vs. bulk) would alter moisture and gas exchange, potentially shaping microbial communities, and (iii) marketability status would reflect differences in initial fruit quality and associated microbiota. Understanding these relationships is critical for designing microbiome-informed strategies to reduce spoilage and improve postharvest quality. This work provides the primary baseline data on pear fungal and bacterial diversity and composition, which can be foundational in the development of effective microbial management strategies. From an industry perspective, understanding the temporal shifts of the pear surface microbiome can inform storage practices, microbial risk assessment, and potential biocontrol strategies by identifying critical windows of community change, predicting conditions that favor pathogen establishment, and guiding interventions that promote beneficial taxa while suppressing spoilage organisms. Such insights enable the development of targeted treatments, optimized storage parameters, and microbiome-informed quality control protocols that reduce postharvest losses and enhance food safety.

## RESULTS

### Alpha diversity

Estimated Chao1 alpha diversity of ITS1 amplicon sequencing variants (ASVs) for fungal communities was notably lower (average 18.3, CI 15.5–21.1) than estimated alpha diversity of 16S ASVs in bacterial communities (average 166.4, CI 136.2–196.6). The diversity observed in the culturable communities was significantly (*P* < 0.01) lower than total rinse communities for both fungi (ITS1) and bacteria (16S). For example, total bacterial communities showed an average of 298 ASVs, while bacterial communities recovered from cultures showed an average of 32 ASVs. The average number of fungal (ITS1) ASVs was 25 for DNA isolated from the total rinse, and 6 for DNA isolated from cultures. No significant differences in alpha diversity of fungal communities were found between bulk and individually wrapped pears. Bacterial communities did show a significant difference (*P* < 0.05) with bulk (182.51 ASVs) having a higher average diversity than wrapped (105 ASVs). No significant differences in Chao1 richness indices were observed when marketable versus unmarketable pears were compared for both fungal and bacterial communities.

Using the total rinse richness estimates, we examined significant differences in richness between the 3-, 6-, and 9-months time points. Bulk and wrapped pears were tested separately. For the fungal communities in bulk pears, we saw no significant change in the Chao1 index between the 3- and 6-months time points, while a significant (*P* < 0.05) decrease was observed from the 6 to 9-months time points. Wrapped pears showed a different pattern; a significant increase in ASV richness was observed between the 3- and 6-months time points, followed by a significant decrease between the 6- and 9-months time points. For bacterial communities in bulk pears, a significant (*P* < 0.05) decrease in richness of ASVs was observed between the 3- and 6-months time points, while a significant (*P* < 0.05) increase was observed between the 6- and 9-months time points. For bacterial communities on wrapped pears, a similar pattern to the observed fungal communities on wrapped pears was observed, with a significant increase in richness between the 3- and 6-months time points, and a significant decrease between the 6- and 9-months time points.

### Beta diversity

**Fig 1:**
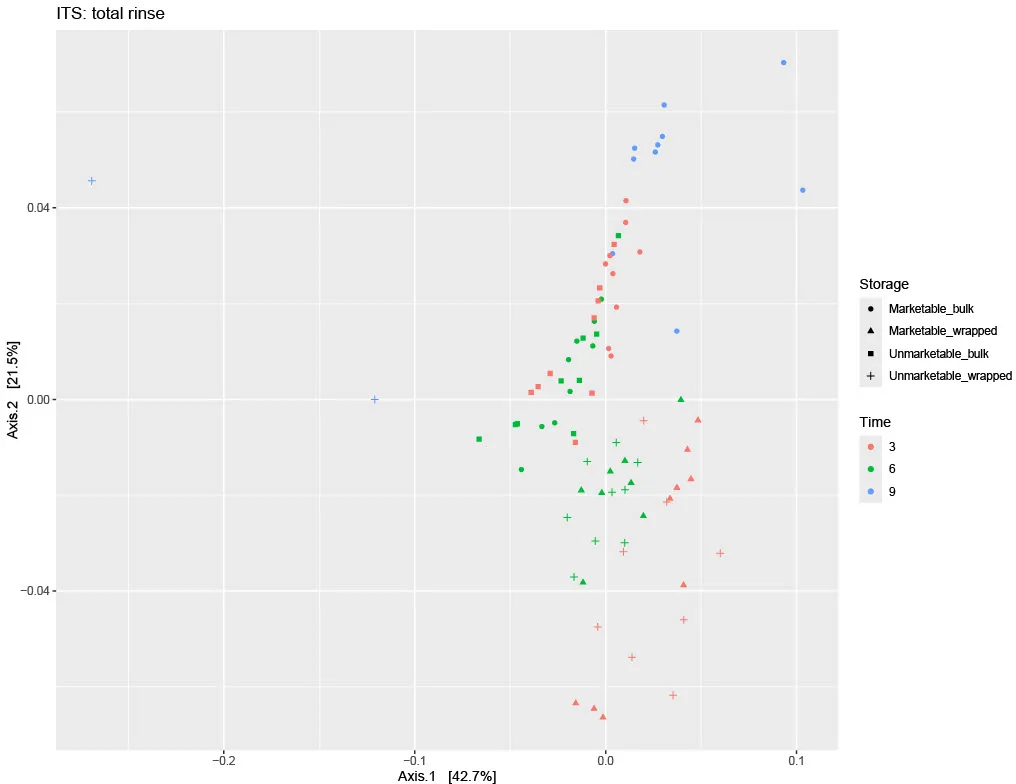
Principal coordinate analysis (PCoA) of fungal (ITS) beta diversity (UniFrac) from pear rinse samples, grouped by storage time (3-, 6-, and 9-month time period), marketability, and packaging condition (wrapped vs bulk).

No distinct clusters could be found for 3- and 6-months fungal communities (Fig. 1), irrespective of marketability or storage condition (bulk or wrapped). Fungal communities of 9-months marketable bulk pears and unmarketable wrapped pears are distinct from the 3- and 6-months pears (Fig. 1). A constrained ordination (CAPscale) analysis based on both bulk and wrapped pears showed that marketability and pears being individually wrapped had a highly significant effect on the beta diversity (*P* < 0.01), while time of sampling had a significant effect at *P* = 0.045. When individually wrapped pears and bulk pears were analyzed separately, time of sampling was still highly significant. Marketability was slightly less significant for bulk pears (*P* = 0.05), while it was not significant for individually wrapped pears (*P* = 0.65).

**Fig 2:**
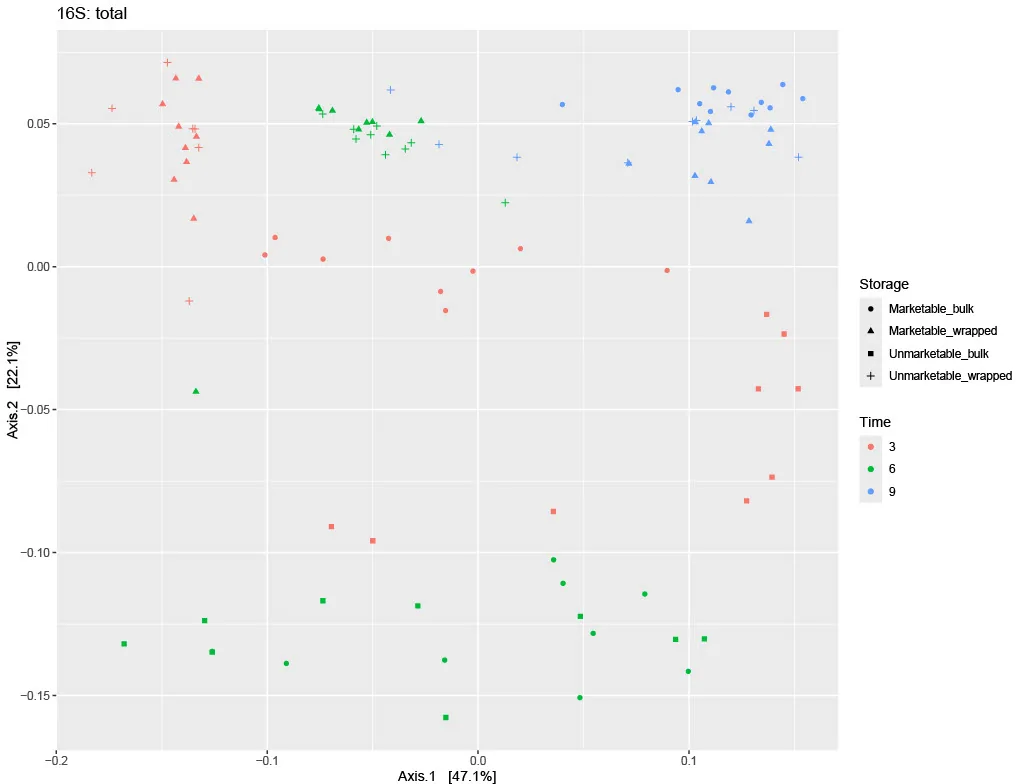
Principal coordinate analysis (PCoA) of bacterial (16S) beta diversity (UniFrac) from pear rinse samples, grouped by storage time (3-, 6-, and 9-months time period), marketability, and packaging condition (wrapped vs bulk).

Principal coordinate analysis (PCoA) of the bacterial communities showed tight clustering by time point for the individually wrapped pears, regardless of marketability (Fig. 2). The 9-months marketable bulk pears cluster with the 9-months individually wrapped pears, while 3- and 6-months bulk pears form very loose clusters that are mainly separated on the y-axis, with no clear clustering by marketability (Fig. 2). CAPscale analyses confirm the same pattern, showing that both time and being either individually wrapped or bulk have a highly significant (*P* < 0.01) effect on the beta diversity of samples, while marketability is not significant (*P* > 0.05).

### Composition of fungal and bacterial communities associated with the surface of pears

**Fig 3:**
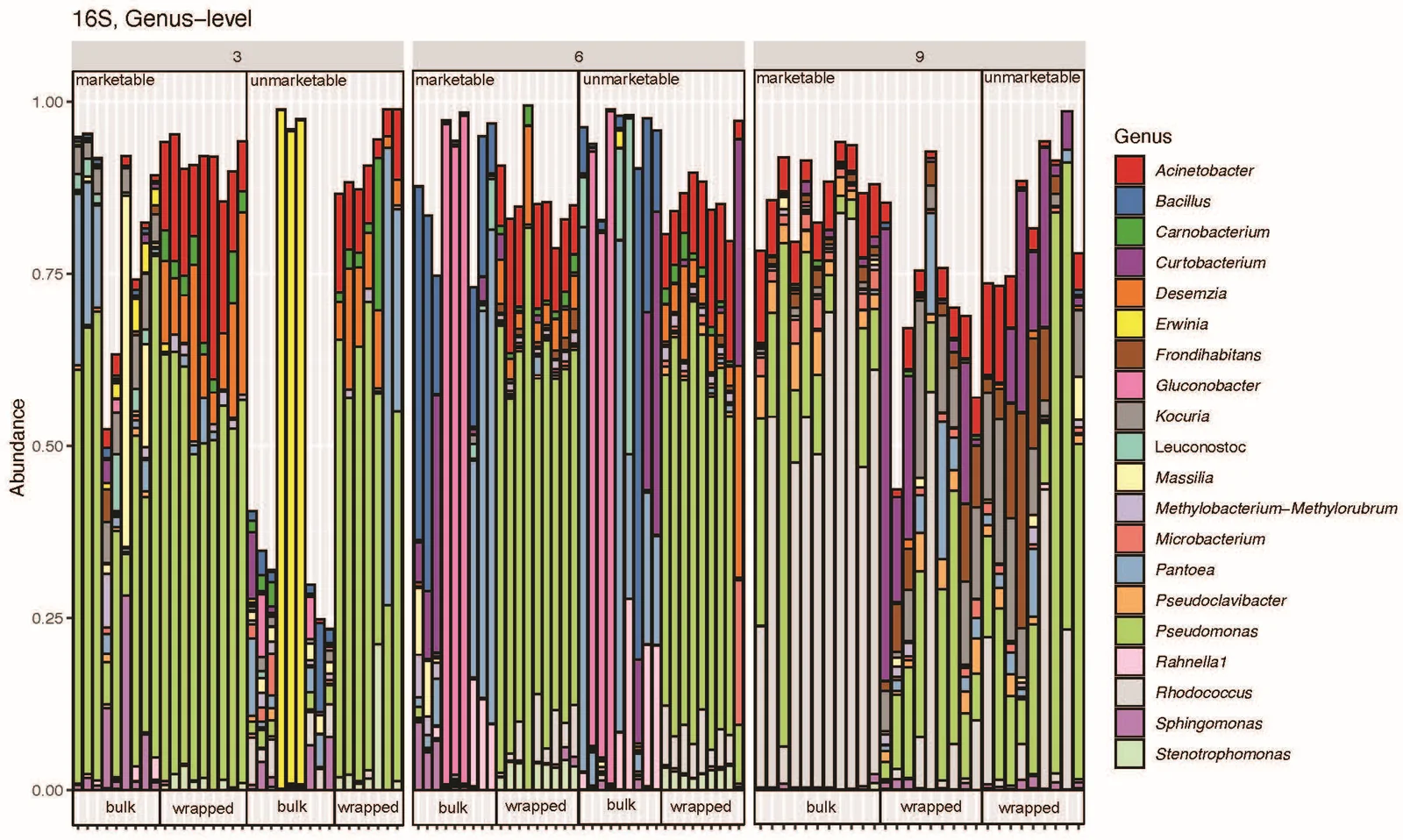
Genus-level composition of bacterial communities on pear rinse surfaces across marketable and unmarketable fruit, stored in bulk or wrapped conditions over 3-, 6-, and 9-month periods.

**Fig 4:**
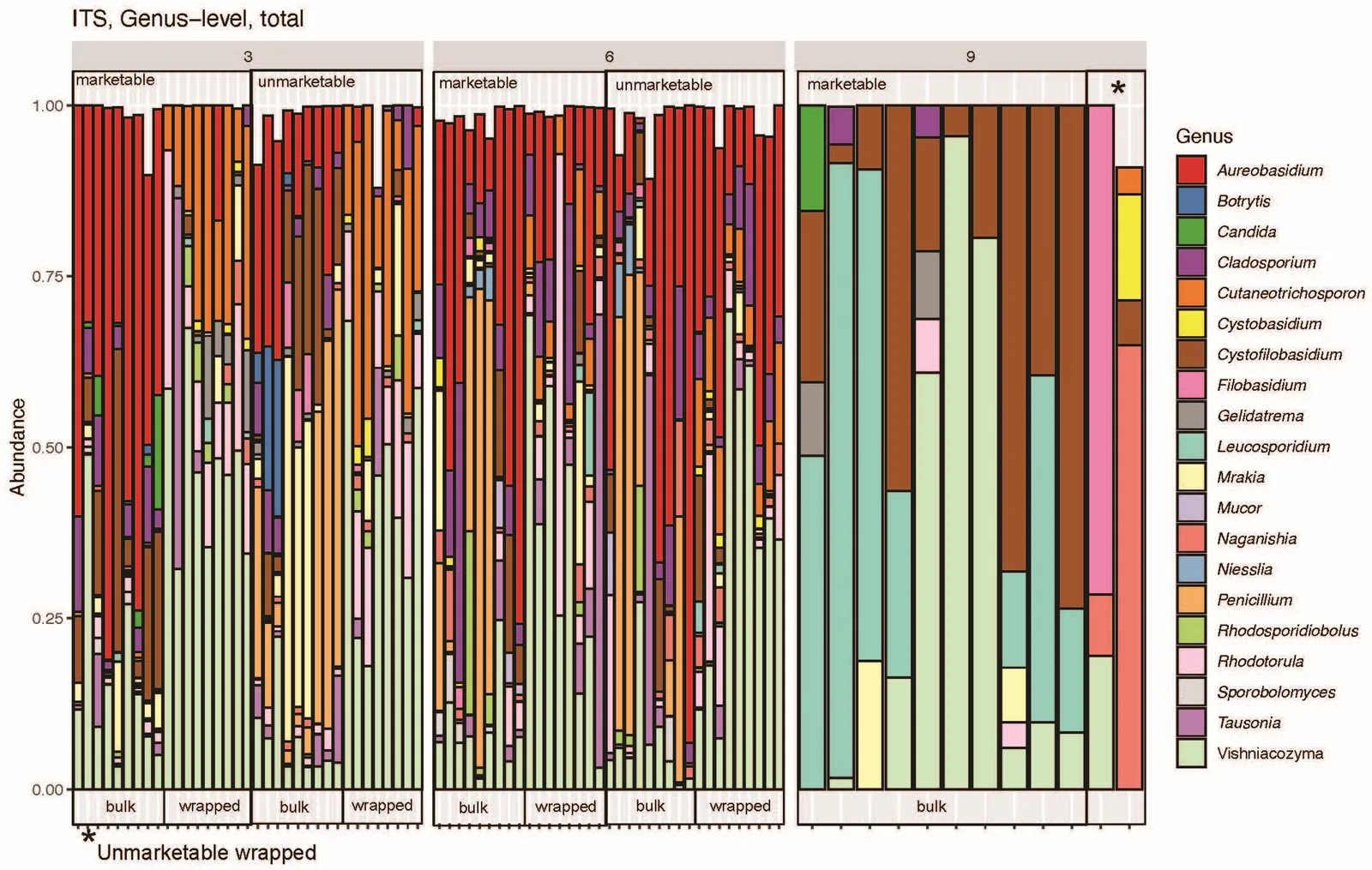
Genus-level composition of fungal communities on pear surfaces across total rinses of marketable and unmarketable fruit, stored in bulk or wrapped conditions over 3-, 6-, and 9-month periods.

The abundance of the bacterial and fungal communities at the genus level obtained from the sequencing analysis of marker genes is shown in Fig. 3 and Fig. 4 respectively. Bacterial communities consisted of a mixture of genera found in food processing environments and other built environments such as *Acinetobacter* (3.31% relative abundance [RA]), *Pseudomonas* (19.2% RA), bacteria associated with the microbiota of plants (e.g., *Frondihabitans* (0.62% RA), *Erwinia* [1.26% RA]), and sugar-rich environments (e.g., *Gluconobacter* [2.41% RA]). The fungal communities (Fig. 4) were dominated at the 3- and 6-months time points by *Aureobasidium* (23.3% RA) and *Penicillium* (9.28% RA) species; however, these genera were virtually absent at the 9-months time point. At all time points, we found a large variety of psychrophilic and psychrotrophic yeasts (e.g., *Mrakia* (2.16% RA), *Leucosporidium* (3.36% RA), *Cutaneotrichosporon* (6.15% RA), *Tausonia* [2.07% RA]). Additionally, fungal genera associated with fruit spoilage were found, such as *Botrytis* (0.33% RA) and *Mucor* (0.14% RA).

For bulk pears, ASVs associated with the bacterial genera *Pantoea* (11.8% RA in overall bulk), *Gluconobacter* (6.83% RA), *Rhanella* (2.31% RA), and *Leuconostoc* (2.35% RA) were significantly enriched in 6-months communities compared to 3-months communities, while *Pseudomonas* in bulk pears (7.40% RA) were significantly reduced. In the 9- versus 6-months comparison, ASVs associated with *Pseudomonas*, *Acinetobacter*, *Microbacter,* and *Rhodococcus* were significantly enriched, while *Gluconobacter* and *Rhanella* were reduced (Fig. 3). For fungal communities in bulk pears, ASVs associated with *Niesslia* (0.34% RA), *Penicillium* (18.60% RA), and *Fusarium* (0.23% RA) were significantly enriched in 6-months communities as compared to 3-months communities, while ASVs associated with *Aureobasidium* (24.29% RA)*, Mrakia* (3.22% RA), *Botrytis* (0.70% RA), and *Candida* (2.77% RA) were significantly reduced (Fig. 4). ASVs associated with *Leucosporidium* (6.80% RA) were significantly enriched in 9-months fungal communities compared to 6-months communities, while ASVs associated with *Aureobasidium*, *Penicillium fuscoglaucum,* and *Neonectria* were significantly reduced.

For individually wrapped pears, ASVs associated with the genera *Williamsia* (0.03% RA), *Pseudomonas* (34.2% RA), *Frondihabitans* (1.25% RA), *Rhodococcus* (2.69% RA), and *Curtobacterium* (6.79% RA) were significantly enriched in 6-months bacterial communities, while ASVs associated with *Pseudomonas* (34.2% RA), *Stenotrophomonas* (0.97% RA), and *Carnobacterium* (1.08% RA) were reduced. In fungal communities in individually wrapped pears, ASVs associated with *Mrakia* (1.55% RA), *Cladosporium* (7.52% RA), and *Vishniacozyma* (31.62% RA) were significantly enriched in 6-months communities, and only one ASV associated with the yeast genus *Metschnikowia* (1.79% RA) was significantly reduced. In 9-months communities compared to 6-months communities, only one ASV associated with the genus *Filobasidium* (1.24% RA) was enriched, while ASVs associated with *Aureobasidium*, *Vishniacozyma*, *Cutaneotrichosporon*, and *Rhodotorula* were among the genera associated with reduced ASVs.

## DISCUSSION

D’Anjou pears are routinely stored for up to 9 months under CA conditions to meet market demands. Limited information exists on pears’ natural microbiota throughout storage. Fungi are important contributors to pear quality, and bacteria can influence not only quality but also the safety of pears. Microbial taxa are commonly characterized using ASVs (20–23). ASVs are often used to estimate species richness, which can then be compared across samples from the environment (e.g., fruits, vegetables, water, or soil). The Chao1 index is a richness estimator that accounts for unseen or rare taxa by incorporating the number of singletons and doubletons (24). In this study, the estimated Chao1 diversity of ITS1 ASVs for fungal communities was notably lower than the estimated diversity of 16S ASVs in bacterial communities. The microbial load on fruits can vary widely depending on the type of fruit, its surface characteristics, growing conditions, and handling (22, 23, 25). Fungal colonization of fruit surfaces tends to be more selective than bacterial colonization (23, 26, 27). Fungal growth on pears, particularly during prolonged storage, is often dominated by a few genera that are well adapted to the fruit environment, such as *Penicillium*, *Botrytis*, or *Alternaria* (28). These fungi can outcompete other taxa through rapid sporulation, enzymatic degradation of fruit tissues, or production of antimicrobial metabolites (29–32), resulting in reduced overall fungal richness. In this study, a large variety of psychrophilic and psychrotrophic yeasts (e.g., *Mrakia*, *Leucosporidium*, *Cutaneotrichosporon*, and *Tausonia*) were observed. *Aureobasidium* and *Penicillium* species were most dominant at 3- and 6-months time points (Fig. 4). *Aureobasidium* is a ubiquitous yeast-like fungus often found on fruit surfaces, known for biofilm formation and tolerance to stress, contributing to long-term survival (33, 34). *Penicillium* is a filamentous fungus responsible for blue and green mold rots on pears and apples, producing enzymes and mycotoxins that drive decay (35, 36). *Botrytis* and *Mucor* were also found (Fig. 3), both spoilage molds that cause gray and soft rot, respectively (37–39). By contrast, bacterial communities on fruit surfaces are typically more diverse, comprising both epiphytic and environmental species, many of which can persist under CA storage without necessarily dominating the community. In this study, the composition of bacterial communities associated with the surface of pears consisted of a mixture of genera found in food processing environments and other built environments (Fig. 3), such as *Acinetobacter*, a genus of Gram-negative bacteria commonly found on plant surfaces and in soil (40). *Pseudomonas*, a diverse group of Gram-negative bacteria frequently isolated from fruits and vegetables, with some species contributing to spoilage through enzymatic degradation and biofilm formation (41, 42), was also detected. Other bacteria associated with the microbiota of plants (e.g., *Frondihabitans*, *Erwinia*) and sugar-rich environments (e.g., *Gluconobacter*) were also found. These findings highlight the importance of tailoring postharvest interventions to the distinct ecological patterns of fungi and bacteria. Because fungal communities are dominated by a few pathogenic taxa, control strategies can focus on targeted approaches such as selective fungicides, biocontrol agents, or packaging technologies that inhibit these specific organisms. In contrast, bacterial communities are more diverse and resilient, requiring holistic management strategies that influence overall community composition rather than single species. Such approaches may include microbiome-informed sanitation, environmental adjustments to storage conditions, or the introduction of beneficial microbes to promote competitive exclusion. Understanding these dynamics enables the development of integrated, microbiome-based interventions that improve pear quality and reduce postharvest losses. Importantly, identifying these dominant genera and tracking how their relative abundance changes over time is critical because such descriptions for pears are scarce. While these taxa are broadly abundant in fruit microbiomes, confirming their dynamics in pears provides a baseline for future research and industry practices. For example, our observation that *Pseudomonas* and *Burkholderia* increased over extended storage aligns with trends reported in apples under CA storage (17), and the persistence of *Penicillium* on unmarketable pears mirrors findings on bacterial viability and pathogen persistence in pears (18, 19). These comparisons reinforce that our results fit within the broader landscape of fruit microbiome research while filling a gap for pears. Reduced oxygen and elevated carbon dioxide are effective at suppressing fungal growth, particularly of aerobic saprophytic fungi such as *Penicillium*, *Botrytis*, and *Mucor* (38–40). Storage conditions may differentially influence fungi and bacteria. This difference underscores the need for targeted strategies: fungal control may require interventions aimed at a few dominant pathogens, while bacterial management must consider a broader, more resilient community, as mentioned previously. Understanding these dynamics is critical for designing microbiome-informed approaches to improve pear quality and reduce postharvest losses.

Reduced oxygen storage is widely recognized as an effective strategy to delay fungal growth and extend fruit shelf life. CA systems typically maintain oxygen levels between 1%–3%, which significantly inhibits pathogens such as *Penicillium* without compromising fruit quality (43, 44). For example, Archer et al. (43) demonstrated that oranges stored under low oxygen (0.9 kPa O₂, ~0.9%) exhibited markedly reduced mold lesion development compared to untreated controls, while key quality attributes remained largely unaffected. Similarly, combining CA storage with biocontrol agents such as antagonistic yeasts has proven effective in apples and pears, where 3% O₂ and elevated CO₂ levels suppressed gray mold and blue mold and enhanced yeast efficacy (44). These findings underscore the importance of oxygen management as a non-chemical intervention for postharvest disease control. By contrast, Hoogerwerf et al. (45) reported an atypical modified atmosphere containing 80% oxygen and 20% CO₂, which inhibited *Botrytis cinerea*, *Penicillium discolor*, and *Rhizopus stolonifer* for up to 17 days, while elevated CO₂ alone suppressed growth for about 11 days. This high-oxygen approach differs fundamentally from conventional CA strategies, which rely on hypoxic conditions, and highlights the diversity of atmospheric manipulations being explored for decay control. Such variability reflects a broader trend toward tailoring storage atmospheres to specific pathogen profiles and fruit physiology rather than relying on a single “one-size-fits-all” approach. However, these treatments may also influence bacterial taxa differently, as genera like *Pseudomonas* and *Acinetobacter* can adapt to modified atmospheres through metabolic flexibility (43–45). This suppression of fungi under CA atmosphere storage could therefore have limited the number of detectable fungal ASVs compared to bacteria, illustrating how atmospheric interventions shape both fungal and bacterial communities.

When comparing communities obtained from culturable samples with those detected in whole-pear rinses, alpha diversity indices consistently showed fewer total ASVs in the culturable fraction. This highlights the value of whole-fruit rinsing approaches and underscores the biases inherent to culture-based methods, which often favor fast-growing or easily culturable taxa prioritized by target growth conditions (e.g., *Pseudomonas*, *Enterobacteriaceae*) while underrepresenting slow-growing, stressed, or obligate symbiotic organisms (13, 14, 46, 47). For example, Ruiz Rodriguez et al. (47) used both culture-dependent and shotgun metagenomic (culture-independent) approaches to characterize microbial communities on tropical fruit surfaces (e.g., guava, papaya, and medlar flowers). They found that while the most abundant species were identified by both methods, less abundant taxa were often missed by culture-based methods, likely due to selective enrichment in cultures. The total rinse approach avoids the direct bias imposed by cultivation media used in a culture-dependent (enrichment) strategy and is recommended in future studies when the objective is to characterize community dynamics irrespective of target organisms or indicators (e.g., generic *Escherichia coli*). However, culture-based methods offer unique advantages, such as enabling isolation of viable organisms for downstream phenotypic characterization, antimicrobial resistance testing, and functional studies, capabilities that sequencing alone cannot provide. Sequencing approaches may detect DNA from non-viable or dead cells, which can complicate interpretation of active microbial populations. Therefore, integrating both culture-dependent and culture-independent approaches simultaneously provides the most comprehensive understanding of microbial communities, balancing taxonomic breadth with functional and viability insights.

Storage placement conditions such as bulk vs individually wrapped had no effect on fungal diversity over time, but bacterial communities showed a significant difference (*P < 0.05*). Wrapping can create microenvironments with altered humidity and gas exchange (48), potentially selecting for specific fungal taxa while excluding others. In bulk storage, fungal spores may disperse more easily but remain dominated by a few highly competitive taxa, leading to relatively low richness. The decline in fungal alpha diversity observed in bulk-stored pears between 6- and 9-months can be explained by both physiological constraints of the storage environment and competitive ecological dynamics among fungal taxa. Prolonged exposure to low O₂ and elevated CO₂ conditions suppresses the growth of many aerobic organisms, creating a selective environment where a subset of storage-adapted fungi, such as *Penicillium*, *Alternaria*, and *Botrytis*, can persist and eventually dominate, as discussed previously (29, 35–38). Such selective pressure likely explains the significant reduction in pear community richness as storage time progresses, particularly after 9 months. While shifts in microbial communities were observed during storage, these changes are not solely attributable to CA conditions. Fruit physiology plays a major role, as ripening, senescence, and associated changes in pH, nutrient availability, and tissue integrity create niches that favor certain taxa. Other factors beyond CA include temperature, humidity, storage duration, and initial microbiome composition, all of which interact to shape community dynamics. However, this study did not explore these aspects, leaving an opportunity for future research to investigate how such factors contribute to microbiome shifts during storage. Therefore, while CA may influence microbial succession by altering oxygen and CO₂ levels, it operates within a broader context of physiological and environmental changes inherent to postharvest storage.

Bacteria can more readily occupy micro-niches across fruit surfaces and persist in a broader range of storage conditions, maintaining higher observed diversity. In the fresh pear industry, wrapping papers impregnated with copper carbonate, antioxidants (e.g., ethoxyquin), and oils have been widely used for over a century to reduce physiological disorders such as superficial scald (5, 49). However, assessment of wrapping paper efficacy on fresh pears has focused exclusively on *B. cinerea* (50), leaving broader microbial impacts largely unexplored. In this study, both wrapped and bulk pears were stored under CA conditions, which are known to suppress many epiphytic taxa, including genera such as *Sphingomonas* and *Methylobacterium*. The early decline in alpha diversity observed in bulk pears between 3- and 6-months may therefore reflect the combined effects of CA conditions and packaging. This distinction is important because while wrapping papers may influence surface chemistry and microenvironment, the overarching atmospheric conditions likely exert a stronger selective pressure on microbial communities. As tissues soften/senesce, microleaks of sugars/phenolics create new niches (51). This can enable secondary colonizers and metabolically flexible taxa (e.g., *Pseudomonas*, *Pantoea/Enterobacteriaceae*, *Acinetobacter*) to rebound, raising richness. Similar patterns have been documented in pears treated with 1-methylcyclopropene (18), a synthetic plant growth regulator. The study reported that fungal pathogens such as *Botryosphaeria*, *Penicillium*, and *Fusarium* were suppressed compared to untreated fruit (23). Apples also showed strong temporal succession in storage, with stable bacterial (*Pseudomonas*, *Pantoea*) and fungal (*Aureobasidium*, *Cladosporium*) cores persisting while other taxa turn over across cold storage (17, 52). Together, these findings highlight that fruit microbiomes are strongly shaped by storage environment and packaging type.

Marketability of fruits typically aligns with standards used to assess consumer acceptability, which are based on visual attributes and not on food safety considerations. This includes blemishes, patches of rot or mold, sun- or frost-burn, bruising, or scarring. The absence of significant differences in alpha diversity between marketable and unmarketable pears suggests that the transition from marketable to unmarketable may not depend on the total diversity of surface-associated microbes, but rather on the activity and behavior of specific taxa. For example, unmarketable pears may reflect microbial processes such as the internalization of microbes and pathogens into fruit tissues (53, 54) or the establishment of biofilms, which would not necessarily be captured by surface-level diversity measures. Therefore, while fruit physiology remains the dominant driver of marketability, microbial dynamics, particularly those associated with spoilage organisms, represent an additional layer of complexity that warrants further investigation.

Fungal communities associated with pears exhibited temporal changes during storage, with the most pronounced shifts occurring at the 9-months time point. These patterns suggest that storage duration interacts with packaging and fruit condition to shape fungal community structure. While statistical analyses confirmed significant effects of packaging and marketability on richness, the broader implication is that postharvest handling practices can influence microbial succession in ways that may affect decay risk and shelf life. Similar trends have been reported in other fruits (55), where packaging and storage conditions alter fungal ecology and pathogen prevalence, reinforcing the need to consider microbiome dynamics in integrated postharvest management strategies. Understanding these interactions could inform interventions that minimize spoilage while maintaining fruit quality, particularly as industry moves toward reduced chemical inputs and extended storage periods. For bacterial communities, ordination analyses demonstrated tighter clustering by storage time for individually wrapped pears, regardless of marketability, indicating that temporal dynamics outweighed quality status (Fig. 2). By contrast, bulk pears at 3- and 6-months exhibited loose clustering, with no clear separation by marketability, whereas 9-months marketable bulk pears clustered closely with 9-months wrapped pears. Together, these findings indicate that while time and packaging are consistent drivers of microbial succession in stored pears, marketability is more relevant for fungal than bacterial communities, particularly in bulk fruit. While this study does not aim to detect foodborne pathogens, understanding the broader microbial ecology of pears during storage can provide context for factors influencing fruit quality and spoilage. These insights complement pathogen-focused research by identifying conditions that may favor beneficial or spoilage-associated taxa, informing strategies to maintain fruit integrity rather than serving as a pathogen surveillance tool.

Our findings support the hypothesis that packaging format is a meaningful driver of the pear microbiome under CA storage, with bulk fruit harboring communities that differ from those on individually wrapped fruit. While temporal change in fruit microbiomes is broadly recognized, our results extend this understanding to pears, a commodity for which such characterization has not previously been reported, and clarify how a practical decision, such as to wrap fruit, can shape community structure in ways relevant to quality, shelf life, and eventually food safety. Although some factors have yielded mixed or null effects, these results still provide value by indicating where operational changes may not deliver microbiome shifts, helping industry prioritize efforts. Importantly, this study was driven by direct industry requests to evaluate packaging and marketability under long-term CA storage, underscoring its applied significance. The microbiome data generated here provide a foundation for multiple practical applications in pear production and postharvest management. First, these profiles can inform strategies to enhance food safety by identifying microbial taxa that may influence the survival or suppression of pathogens on pear surfaces. Second, understanding the native microbiota enables targeted interventions to reduce spoilage organisms, thereby improving shelf life and reducing food waste. Third, these insights can guide the development of biocontrol approaches that leverage beneficial microbes to maintain fruit quality. Finally, the baseline data presented here support future functional studies aimed at elucidating microbial interactions and their role in pear physiology, which could lead to innovations in sustainable production and storage practices. Although the factors examined in this study may seem operational, they represent key decision points in commercial storage and distribution. Microbiome shifts linked to storage duration can inform shelf life predictions, while packaging effects may guide interventions to minimize pathogen persistence. Differences between marketable and unmarketable fruit highlight how initial quality influences microbial ecology, which has implications for sorting and waste reduction strategies.

At the same time, we emphasize key limitations that temper interpretation and point to next steps. First, longitudinal sampling was not performed on the same lot, so apparent temporal patterns should be viewed as preliminary indications rather than definitive trajectories; future work should track repeated samples from a single lot to confirm these trends. This limitation is inherent to microbiome research conducted in industrial and high-throughput fruit systems, which is the challenge of tracking the same microbial batch over time. In commercial fruit production and packing environments, products move rapidly through processing lines, are exposed to fluctuating environmental conditions, and undergo routine sanitation, making it difficult to repeatedly sample identical microbial communities across production cycles. Similar constraints have been reported in longitudinal microbiome studies of tree fruit packing facilities, where microbial community composition varied substantially across seasons and facility locations despite standardized sampling approaches (56). Likewise, comprehensive characterizations of environmental microbiota in fruit packing environments have demonstrated strong temporal and spatial variability (57), emphasizing that cross-sectional or batch-based sampling may not fully capture the dynamic nature of fruit surface microbiomes in industrial settings. These findings align with our observations and underscore that microbial communities on fruit surfaces are highly responsive to operational practices, environmental inputs, and temporal changes. While this approach cannot confirm true longitudinal shifts, it provides a preliminary indication of how microbial communities may evolve during extended storage. Future studies should incorporate repeated sampling from the same lot to validate these trends. Additionally, amplicon sequencing captures only a subset of the microbial community, potentially overlooking functionally important but low-abundance taxa or those with atypical marker genes. This limitation is common in microbiome studies and highlights the need for complementary approaches such as shotgun metagenomics (15). Another important consideration is that while diversity and community composition were characterized, functional implications, such as metabolite shifts, pathogen suppression, or interactions with fruit physiology, were not directly tested. Microbiome data alone cannot predict whether observed taxa contribute to spoilage or confer protective effects against pathogens. Future research should therefore integrate multi-omics approaches, including metabolomics and transcriptomics, to link microbial succession with biochemical changes during storage. Experimental inoculations under controlled conditions could further clarify causal relationships between microbiome composition and outcomes such as decay incidence or patulin accumulation, as demonstrated in other studies (10, 11, 58). Finally, we acknowledge that field effects can influence the fruit microbiome; however, the specific sourcing information regarding whether pears originated from a single farm or multiple farms was not disclosed to us by the industry partner, and therefore this level of field-level detail was not available for inclusion in the manuscript. Further work may include longitudinal characterization of the pear microbiome from harvest through postharvest handling and storage to better understand how microbial communities shift throughout the commercial supply chain.

### Conclusion

In conclusion, no significant differences in Chao1 alpha diversity index were observed in the overall bacterial and fungal community structures between marketable and unmarketable pears, despite the presence of known spoilage fungi such as *Botrytis*, *Mucor*, and *Penicillium*. Instead, storage practices played a more prominent role, with packaging type (individually wrapped vs bulk) significantly shaping the composition and succession of both fungal and bacterial communities. For fungi, alpha diversity peaked at 6 months before declining at 9 months, while bacterial communities from individually wrapped pears followed a similar temporal trend. In contrast, bulk pears only showed a significant decline in bacterial diversity between 6 and 9 months. These findings underscore the critical influence of storage conditions on microbiome dynamics and highlight the need to consider microbial ecology in postharvest management strategies. By providing the first comprehensive baseline of pear-associated microbiomes under commercial storage, this study lays the foundation for future research aimed at linking microbial succession with fruit physiology, spoilage risk, and food safety. Such knowledge is essential for developing microbiome-informed interventions that optimize storage practices, reduce losses, and enhance the quality and safety of pears in the supply chain.

## MATERIALS AND METHODS

### Pears

D’Anjou pears were shipped from an industry collaborator in Washington State to the Center for Food Safety (Griffin, GA) at 3, 6, and 9-months after harvesting. All pears were stored under identical CA conditions (temperature, humidity, and gas composition) in the same storage room by the collaborator in Washington State. CA atmosphere was set at 2%–3% O_2_ and 1% CO_2_ and kept at 3°C. Marketable and unmarketable pears were packaged in separate bags, and unmarketable pears–normally discarded or processed–were kept in their bags specifically for this study. Pears (*N* = 256) were either received in bulk or individually wrapped; for both categories, pears were marked as "marketable" or "unmarketable," as determined by industry collaborators. Marketability typically aligns with standards used to assess consumer acceptability, which are based on visual attributes, including the absence of visible defects (e.g., bruising, decay, and scarring), uniform size and shape, and acceptable firmness and color; pears not meeting these criteria were classified as unmarketable considerations. Marketable and unmarketable pears were collected for each time point and type of pear, with the exception of bulk 9-months pears, for which only marketable fruit was obtained. Pears were wrapped with tissue paper containing copper carbonate (1.3%) and ethoxyquin (0.1%), per industry practices. Individually wrapped pears were not sampled at 9-months because these samples were not provided by the collaborator at that time point. This was due to logistical constraints in sample availability rather than a methodological decision.

### Bacterial and fungal isolation

Five pears per lot of the same marketability type were sonicated with 250 mL of wash solution comprising 1× Tris-EDTA buffer (G-Biosciences, Saint Louis, MO, USA) supplemented with 2% Tween 80 (Research Products International, Mount Prospect, IL, USA) in a stomacher bag. To dislodge microorganisms from the surface of the samples, one individual pear was sonicated for 1 min, for a total of five pears/minutes (VWR International, LLC, Radnor, PA, USA). During sonication, samples were securely contained within sterilized sampling bags to maintain aseptic conditions. To recover the culturable microbiota, 10 mL of the sample was collected, and 4 mL aliquots were plated in duplicate onto Tryptic Soy Agar (TSA; Difco, Becton Dickinson Co, Franklin Lakes, NJ, USA). The TSA plates were incubated at 35°C for 24 h to allow bacterial growth. For culturable fungi, 1 mL of the same (4 mL) aliquot was plated in duplicate onto Potato Dextrose Agar (PDA; Difco, Becton Dickinson Co), and the plates were incubated at 25°C for 7 days.

### DNA isolation of microbial communities of the pear surface

The remaining wash solution (~235 mL) was divided into seven aliquots of 30–35 mL in sterilized conical centrifuge tubes. These aliquots were centrifuged at 3,180 × *g* for 15 min at 4°C. Following centrifugation, the clear supernatant was carefully removed, leaving a dense pellet in each aliquot. The pellets from all aliquots were combined into a single tube and centrifuged again at 3,180 × *g* for 15 min at 4°C. After discarding the supernatant, the consolidated pellet was transferred to a microcentrifuge tube and subjected to a final centrifugation at 10,000 × *g* for 10 min. The remaining concentrated pellet served as the sample for DNA extraction.

For the culturable microbiota, 1 mL of Phosphate Buffered Saline (PBS; Difco, Becton Dickinson Co) was pipetted onto the TSA and 5 mL of PBS onto the PDA plate surface, and the bacterial lawn from each of the plates was gently removed using a sterile L-shaped spreader. The resulting suspension was transferred into 2 mL tubes for each sample. The pooled solution was then concentrated by centrifugation at 10,000 × *g* for 5 min at 4°C and stored at −20°C until DNA extraction. DNA was extracted from the pellet using the DNeasy PowerSoil Kit (Qiagen, Hilden, Germany) in accordance with the manufacturer’s instructions.

### Amplicon sequencing of bacterial and fungal communities

High-throughput sequencing was performed on the Illumina MiSeq sequencer at the University of Georgia’s Center for Food Safety, generating paired-end 300 bp reads for each amplicon. To target bacterial communities, the V3–V4 region of 16S rRNA was sequenced, while the first internal transcribed spacer (ITS1) of the eukaryotic ribosomal cistron was targeted for the fungal communities. The bacterial community was characterized by amplifying the V3–V4 regions of the 16S rRNA gene using primers 341F (CCTACGGGNGGCWGCAG) and 785R (GACTACHVGGGTATCTAATCC), as recommended in the Illumina 16S Metagenomic Sequencing Library Preparation Guide for generating a ~460  bp amplicon with optimal genus-level resolution. Fungal communities were profiled by targeting the ITS1 region with the ITS1f–ITS2 primer pair (e.g., CTTGGTCATTTAGAGGAAGTAA and GCTGCGTTCTTCATCGATGC), following the Illumina ITS Metagenomics Protocol, which ensures accurate amplification and sequencing of the fungal mycobiome (59). For this study, the SILVA database was used for classification: SILVA 138.2 NR99 (60). The generated reads were paired-end reads, and the metadata and raw reads can be found at PRJNA1426429.

### Bioinformatics and statistical analyses

All bioinformatics and statistical analyses, with the exception of sequence alignment and phylogenetic inference, were performed in R 4.3.3 (61). Sequence alignment was performed using MUSCLE 5.1 (62), and phylogenetic trees were inferred using FastTree 2.1.9 (63) on a Linux server. The R package DADA2 (64) was used to infer amplicon sequence variants, largely following the tutorials outlined on the authors’ website (65). Paired-end reads (forward and reverse) were merged using DADA2 to generate high-quality consensus sequences of the V3–V4 region prior to sequence alignment and taxonomic classification, ensuring accurate reconstruction of the full amplicon (~460 bp).

Phyloseq (66) was used for further microbiome analyses, largely following the workflow outlined in (67). DESeq2 analyses for individual time points were performed separately for bulk and individually wrapped pears to infer which ASVs were enriched in the communities at different time points. Overrepresented taxa in pairwise comparisons (e.g., month 3 versus 6, marketable versus unmarketable) were also inferred using the DESeq2 (1.40.2) R package (68), while CAPscale analyses in the vegan R package (69) were performed to infer which parameters were significantly influencing beta diversity in the microbial communities.

Alpha diversity of amplicon sequence variants (ASVs) was estimated using the Chao1 statistic. The Chao1 index is widely used in microbiome studies, including food systems, because it estimates species richness while accounting for undetected rare taxa, which is critical in environments where sequencing depth may miss low-abundance organisms. This helps researchers avoid underestimating diversity and provides a more accurate baseline for comparing treatments or conditions (20, 70, 71). Alpha diversity indices were compared between two groups (e.g., marketability, packaging type, total rinse vs culturable) using Welch’s *t*-test, which accounts for unequal variances. To assess changes in alpha diversity over time, ANOVA followed by Tukey’s post hoc test was used. To compare the composition (beta diversity) of the fungal and microbial communities found on the pear surfaces, PCoA were performed based on weighted UniFrac (72) distances. To test which parameters (e.g., marketability, bulk versus wrapped) were significantly associated with beta diversity, we used a constrained ordination via CAPscale analysis in the vegan package (2.7.1). Beta diversity analyses were only performed for the ‘total rinse’ samples, as the data sets of culturable organisms only represent small subsets of the total diversity. *P <* 0.05 was considered significant. The parameters and workflow used can be found in the R code at https://github.com/hcdenbakker/Pears_16S_ITS (73).

## Data Availability

Raw data are available at the following NCBI project: PRJNA1426429. A repository for the R code and additional data can be found at https://github.com/hcdenbakker/Pears_16S_ITS.
